# Pharmacotherapy for alcohol use disorder among adults with medical disorders in Sweden

**DOI:** 10.1186/s13722-024-00471-9

**Published:** 2024-05-19

**Authors:** Anastasia Månsson, Anna-Karin Danielsson, Hugo Sjöqvist, Toivo Glatz, Andreas Lundin, Sara Wallhed Finn

**Affiliations:** 1 Department of Global Public Health, Karolinska Institutet, Stockholm, 171 77 Sweden; 2 Charité – Universitätsmedizin Berlin, corporate member of Freie Universität Berlin and Humboldt-Universität zu Berlin, Institute of Public Health, Charitéplatz 1, Berlin, 10117 Germany; 3 Centre for Epidemiology and Community Medicine, Stockholm Region, Stockholm, 171 77 Sweden; 4 Mottagningen för alkohol och hälsa, Stockholm Center for Dependency Disorders, Health Care Services, Riddargatan 1, 114 35, Stockholm, Sweden; 5 Unit of Clinical Alcohol Research (UCAR), University of Southern Denmark, J.B. Winsløws Vej 20, entrance. 220 B, Odense, 5000 Denmark

**Keywords:** Alcohol use disorder, Epidemiology, Pharmacotherapy, Alcohol-attributable medical comorbidities, Sweden

## Abstract

**Background:**

Alcohol-attributable medical disorders are prevalent among individuals with alcohol use disorder (AUD). However, there is a lack of research on prescriptions of pharmacological treatment for AUD in those with comorbid conditions. This study aims to investigate the utilization of pharmacological treatment (acamprosate, disulfiram and naltrexone) in specialist care among patients with AUD and comorbid medical diagnoses.

**Methods:**

This was a descriptive register-based Swedish national cohort study including 132,728 adults diagnosed with AUD (*N* = 270,933) between 2007 and 2015. The exposure was alcohol-attributable categories of comorbid medical diagnoses. Odds ratios (OR) were calculated using mixed-effect logistic regression analyses for any filled prescription of acamprosate, disulfiram or oral naltrexone within 12 months post AUD diagnosis.

**Results:**

Individuals with comorbid alcohol-attributable medical diagnoses had lower odds of filling prescriptions for any type of AUD pharmacotherapy compared to those without such comorbidities. Cardiovascular (OR = 0.41 [95% CI: 0.39–0.43]), neurological (OR = 0.52 [95% CI: 0.48–0.56]) and gastrointestinal (OR = 0.57 [95% CI: 0.54–0.60]) diseases were associated with the lowest rates of prescription receipt. The presence of diagnoses which are contraindications to AUD pharmacotherapy did not fully explain the low prescription rate.

**Conclusion:**

There is a substantial underutilization of AUD pharmacotherapy in patients with AUD and comorbid medical disorders in specialist care. Increasing the provision of pharmacotherapy to this group of patients is essential and may prevent morbidity and mortality. There is a need to further understand barriers to medical treatment both from the patient and prescriber perspective.

**Supplementary Information:**

The online version contains supplementary material available at 10.1186/s13722-024-00471-9.

## Background

Alcohol use is linked to a wide range of diseases, including both psychiatric such as alcohol use disorder (AUD) and depression and medical conditions such as liver diseases and cardiovascular diseases [1]. Globally, approximately 100 million individuals are affected by AUD [2], with a prevalence of alcohol dependence of about 4% in Sweden [3]. Psychiatric and medical comorbidities are prevalent among individuals with AUD [4]. However, heavy alcohol use is associated with low treatment seeking to primary care and shorter hospital stays [5], which can negatively impact health outcomes of co-occurring disorders. Consequently, treatment for AUD may be particularly important for people with comorbid medical diseases.

Four pharmacological agents are currently approved by the Swedish Medical Products Agency (Läkemedelsverket) and by the European Medicines Agency (EMA) for treatment of AUD: acamprosate, disulfiram, naltrexone and nalmefene. Acamprosate, disulfiram and naltrexone received the highest recommendation from the Swedish National Board of Health and Welfare, meaning they should be offered to all patients with AUD in health care services [6]. Nalmefene has a lower recommendation and can be offered. However, nalmefene is not part of the subsidised prescribed pharmacotherapies in the national health care coverage, and seldomly prescribed [7]. In Sweden, AUD is the only approved indication for these four pharmacological treatments.

Despite the demonstrated efficacy and availability of pharmacotherapy for AUD treatment [8], its utilization in clinical practice remains scarce. Studies from Australia, England and the United States indicate that only 3–12% of patients with AUD receive pharmacotherapy [9–11], while in Sweden, the proportion is comparatively higher at 23–24% [7].

Existing literature suggests that females, individuals younger than 55 years old, and those residing in urban areas are more likely to receive AUD pharmacotherapy [7, 11–14]. Moreover, individuals with comorbid psychiatric disorders show a higher likelihood of receiving pharmacotherapy for AUD [7, 15]. However, only a limited number of studies have focused on individuals with comorbid medical diagnoses, often including only a narrow range of diagnoses [11, 15]. A recent Swedish study found that concurrent medical diagnoses were associated with markedly lower odds of AUD prescriptions [7]. However, it is not known whether the prescription rates vary between different alcohol-attributable medical disease categories. In order to improve clinical practice, there is a need to better understand the utilization of pharmacological AUD treatment in individuals with concurrent medical disorders.

### Aims

The aim of this study was to investigate the utilization of pharmacological treatment for AUD among patients with comorbid alcohol-attributable medical diagnoses in specialist care.

The specific questions were:

1. What are the utilization rates of pharmacological treatment for AUD within strata of alcohol-attributable medical comorbidities?
2. What are the utilization rates of acamprosate, disulfiram and naltrexone among patients with AUD and alcohol-attributable medical comorbidities?
3. To what extent is the utilization associated with concurrent comorbid diagnoses that are contraindications for pharmacotherapies for AUD?

## Methods

### Study design and data source

This is a register-based longitudinal open cohort design, using data from a linkage of several national Swedish population registers based on personal identification numbers. The registers include officially registered population of Sweden, including migrants with resident permits, born 1932 and later.

### Study population

The cohort was defined as adults, aged 18 and above (born 1932–1997), diagnosed with AUD, excluding acute alcohol intoxication (ICD-10 codes: F10.1-F10.9) between 2007 and 2015 according to the National Patient Register (Supporting Information). The National Patient Register includes diagnoses from specialist out- and inpatient care, but not primary care. The index date was the date of the respective incident AUD diagnosis. The individuals were followed up for 12 months after the diagnosis, concluding on the latest date of 2016-12-31. In cases where patients received multiple AUD diagnoses during the study period, only those diagnoses separated by more than 12 months were included in the analyses. Individuals who died or emigrated within the 12-month follow-up period post AUD diagnosis were removed. A total of 132,728 individuals with AUD were identified during the study period.

### Outcome

The outcome variables in this study were defined as one or more filled prescription of acamprosate, disulfiram or (oral) naltrexone in specialist care within 12 months following the AUD diagnosis, as recorded in the Swedish Prescribed Drug Register. Outcome variables were analysed binary, categorized as “yes” or “no”. Only data on prescriptions picked up in a pharmacy were available for analysis.

### Exposures

Data on medical comorbidity were obtained from the National Patient Register and the National Cancer Register. Comorbidity was defined as one or more in- or outpatient care events with a diagnosis recorded within 12 months following the AUD diagnosis, ensuring clinical relevance for the prescription of AUD medication. Medical comorbidities were identified using ICD-10 codes and included diagnoses attributable to alcohol use [1], as those are highly relevant for individuals with AUD. First, medical diseases were categorized into six groups [1]: cardiovascular diseases, gastrointestinal diseases, diabetes mellitus, neurological diseases, infectious diseases and cancers (Supporting Information). Second, diseases were further categorized into conditions that are contraindications for pharmacological treatment for AUD (disulfiram and naltrexone) and those that are not, according to the drug information provided by the Swedish Association of the Pharmaceutical Industry (Supporting Information). Data on diagnoses of kidney diseases, which are contraindications for acamprosate and naltrexone, were not available in the register linkage, and therefore not included in the analysis.

### Covariates

Based on previous literature, the following covariates were included: sex, age, income, education, domicile, family constellation and country of birth [7, 11–14]. Sex was categorized as male and female, according to the Register of the Total Population (RTB). Age was extracted from the RTB and categorized into five groups: (1) 18–30, (2) 31–45, (3) 46–55, (4) 56–65, and (5) ≥ 66 years old. Household disposable income was extracted from the Longitudinal Integration Database for Health Insurance and Labour Market Studies (LISA) and calculated as the total annual household income and public benefits earned by all family members after taxation. The income variable was categorized into quintiles, with the first quintile representing the lowest income category. Data on education was obtained from the LISA and categorized as follows: (1) < 9 years (= compulsory school in Sweden), (2) 12–13 years (upper secondary school in Sweden), and (3) > 13 years (higher education). Data on domicile was extracted from the RTB and categorized according to population size in the area: large cities (at least 200,000 inhabitants, including Stockholm, Gothenburg and Malmö), medium-sized towns (at least 50,000 inhabitants) and the remaining areas fell into the small town/rural area category. Family constellation was a categorical variable obtained from LISA and grouped into: (1) married/living with partner, (2) living without partner (including unmarried, divorced/separated partner, and widowed/remaining partner). Country of birth were extracted from the RTB and grouped into: (1) Sweden, (2) Nordic countries (Finland, Norway, Denmark and Iceland), (3) North America, Oceania and Europe, and (4) Asia, Africa and South America. For all covariates, the first observation during the study period was used. In unadjusted models, all observations were used, while in adjusted models, only complete cases were considered. Missing data on covariates were present for 0.7% of the cohort (*N* = 2,118).

### Statistical analysis

Descriptive statistics were used to calculate the total numbers of AUD diagnoses by groups of comorbid diagnoses and receipt of AUD pharmacotherapy prescription. Regression models were then used to calculate the relationship between comorbid diagnosis and prescription receipt. In the main regression model, univariate mixed-effect logistic regression analyses were conducted to estimate crude odds ratios (OR) for the association between prescription receipt and comorbid diagnosis groups [16]. We estimated a mixed-effect logistic regression model with comorbid diagnosis alone (univariate model) and comorbid diagnosis and covariates including sex, age, education, income, country of origin, domicile and family constellation (multivariate model) as categorical predictors, and a random intercept by individual pseudo-anonymised patient’s identification number with the independent variance-covariance structure.

First, the analyses were performed using any pharmacotherapy as the outcome. Second, separate analyses were conducted for each pharmacotherapy (acamprosate, disulfiram and naltrexone). Third, we repeated the regression models examining any contraindication, contraindication to disulfiram, contraindication to oral naltrexone, and no contraindication as the exposure of interest.

ORs with 95% confidence interval (CI) were calculated. All analyses were carried out using Stata BE-Basic 17.0. The analyses were not pre-registered, and the results should be considered exploratory.

## Results

Between 2007-01-01 and 2015-12-31, a total of 270,933 AUD diagnoses were recorded for 132,728 individuals. The sociodemographic characteristics of the first (incident) record, i.e., AUD diagnosis, in the study population are reported in Table 1. Out of 132,728 unique individuals diagnosed with AUD, 68.9% were male. Among patients with AUD who had at least one co-occurring medical disorder, a higher proportion were men (75.0% vs. 67.3%). Additionally, a greater percentage of these patients were older than 56 years (59.5% vs. 31.3%) and in the middle-income group (30.8% vs. 24.1%), compared to AUD patients without medical comorbidities.

**Table 1:** Demographic characteristics of patients with AUD at their first AUD diagnosis during the study period

	All patients with AUD		Patients with AUD and medical comorbidities		Patients with AUD without medical comorbidities
	N (%)		N (%)		N (%)
Total	132,728		28,288		104,440
Sex					
Male	91,476 (68.9)		21,229 (75.0)		70,247 (67.3)
Female	41,252 (31.1)		7,059 (25.0)		34,193 (32.7)
Age					
18–30	21,762 (16.4)		1,543 (5.5)		20,219 (19.4)
31–45	29,924 (22.5)		3,708 (13.1)		26,216 (25.1)
46–55	31,551 (23.8)		6,224 (22.0)		25,327 (24.3)
56–65	29,610 (22.3)		8,481 (30.0)		21,129 (20.2)
> 66	19,881 (15.0)		8,332 (29.5)		11,549 (11.1)
Income (percentile)					
0–20	14,792 (11.1)		1,701 (6.0)		13,091 (12.5)
21–40	30,878 (23.3)		7,410 (26.2)		23,468 (22.5)
41–60	32,025 (24.1)		8,723 (30.8)		23,302 (22.3)
61–80	28,375 (21.4)		5,605 (19.8)		22,770 (21.8)
81–100	26,658 (20.1)		4,849 (17.1)		21,809 (20.9)
Education					
≤ 9 years (Compulsory school)	47,190 (35.6)		10,721 (37.9)		36,469 (34.9)
12–13 years (Upper secondary school)	63,268 (47.7)		13,352 (47.2)		49,916 (47.8)
> 13 years (Higher education)	21,210 (16.0)		3,917 (13.8)		17,293 (16.6)
Missing	1,060 (0.8)		298 (1.1)		762 (0.7)
Family constellation					
Married / living with partner	30,898 (23.3)		7,621 (26.9)		23,277 (22.3)
Living without partner	101,685 (76.6)		20,641 (73.0)		81,044 (77.6)
Missing	145 (0.1)		26 (0.1)		119 (0.1)
Country of birth					
Sweden	114,208 (86.0)		24,150 (85.4)		90,058 (86.2)
Nordic countries	8,947 (6.7)		2,503 (8.8)		6,444 (6.2)
Europe, North America, Oceania	5,265 (4.0)		1,005 (3.6)		4,260 (4.1)
Asia, Africa, Middle East, South America	4,173 (3.1)		609 (2.2)		3,564 (3.4)
Missing	135 (0.1)		21 (0.1)		114 (0.1)
Domicile					
Big city	57,025 (43.0)		11,012 (38.9)		46,013 (44.1)
Medium-sized town	52,327 (39.4)		11,836 (41.8)		40,491 (38.8)
Rural area	23,220 (17.5)		5,412 (19.1)		17,808 (17.1)
Missing	156 (0.1)		28 (0.1)		128 (0.1)

The prevalence of alcohol-attributable medical diseases 12 months following an AUD diagnosis was 23.6% (Table 2). Among these, gastrointestinal (10.2%) and cardiovascular (9.2%) conditions were the most prevalent, followed by neurological disorders (4.5%) and diabetes mellitus (3.5%). Comorbid infectious diseases (1.3%) and cancer diagnoses (0.5%) showed the lowest prevalence. Additionally, the prevalence of comorbid medical disorders that were a contraindication for receiving a prescription for at least one AUD medication was 18.1%. One in six, or 15.9%, of AUD diagnoses with concurrent comorbid medical disorders had a filled AUD pharmacotherapy prescription. Comparably, 11.6% of AUD diagnoses with concurrent comorbid disorders with contraindications for disulfiram and naltrexone had a filled prescription (Tables 1 and 2). The proportions of AUD diagnoses with filled prescriptions were similar across the different types of pharmacotherapies (Supplement Table 1).

**Table 2:** Total number of AUD diagnoses by groups of comorbid diagnoses and receipt of AUD pharmacotherapy prescription (*N* = 270,933)

	Any prescriptions		
Alcohol-attributable comorbidities	Yes (%)	No (%)	Total (%)
Any medical comorbidity	10,179 (15.9)	53,915 (26.1)	64,094 (23.7)
Gastrointestinal diseases	4,415 (6.9)	23,180 (11.2)	27,595 (10.2)
Cardiovascular diseases	3,286 (5.1)	21,544 (10.4)	24,830 (9.2)
Neurological diseases	1,806 (2.8)	10,426 (5.0)	12,232 (4.5)
Diabetes mellitus	1,621 (2.5)	7,871 (3.8)	9,492 (3.5)
Infectious diseases	638 (1.0)	2,922 (1.4)	3,560 (1.3)
Cancers	202 (0.3)	1,171 (0.6)	1,373 (0.5)
Contraindication to disulfiram and naltrexone	7,413 (11.6)	41,585 (20.1)	48,998 (18.1)
Contraindication to disulfiram	6,358 (9.9)	38,100 (18.4)	44,458 (16.4)
Contraindication to naltrexone	4,252 (6.6)	20,806 (10.1)	25,058 (9.2)
No contraindication	3,707 (5.8)	15,415 (7.4)	19,122 (7.1)
Total (%) *	63,984 (23.6)	206,949 (76.4)	270,933 (100)

Note: * One patient with AUD can have multiple medical diagnoses

**Fig. 1:**
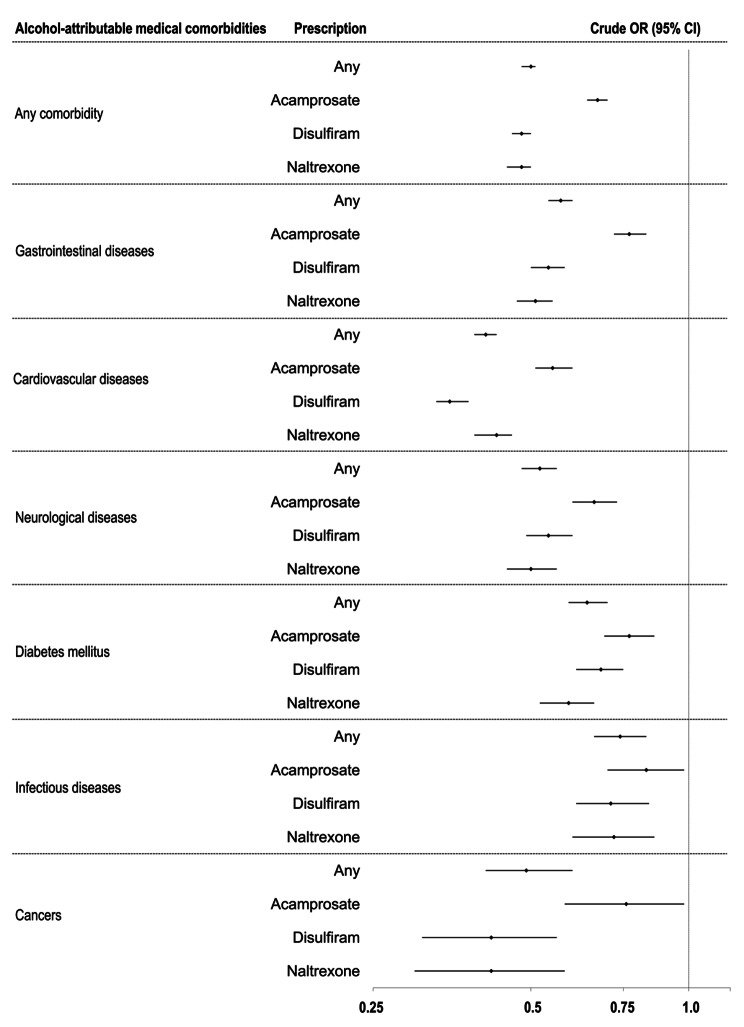
Crude odds ratios for receiving pharmacotherapy prescriptions for alcohol use disorder (AUD) by groups of alcohol-attributable comorbid medical diagnoses (*N* = 270,933). **Note**: OR = odds ratio. CI = confidence interval. For detailed information, see Supplement Table 2

**Fig. 2:**
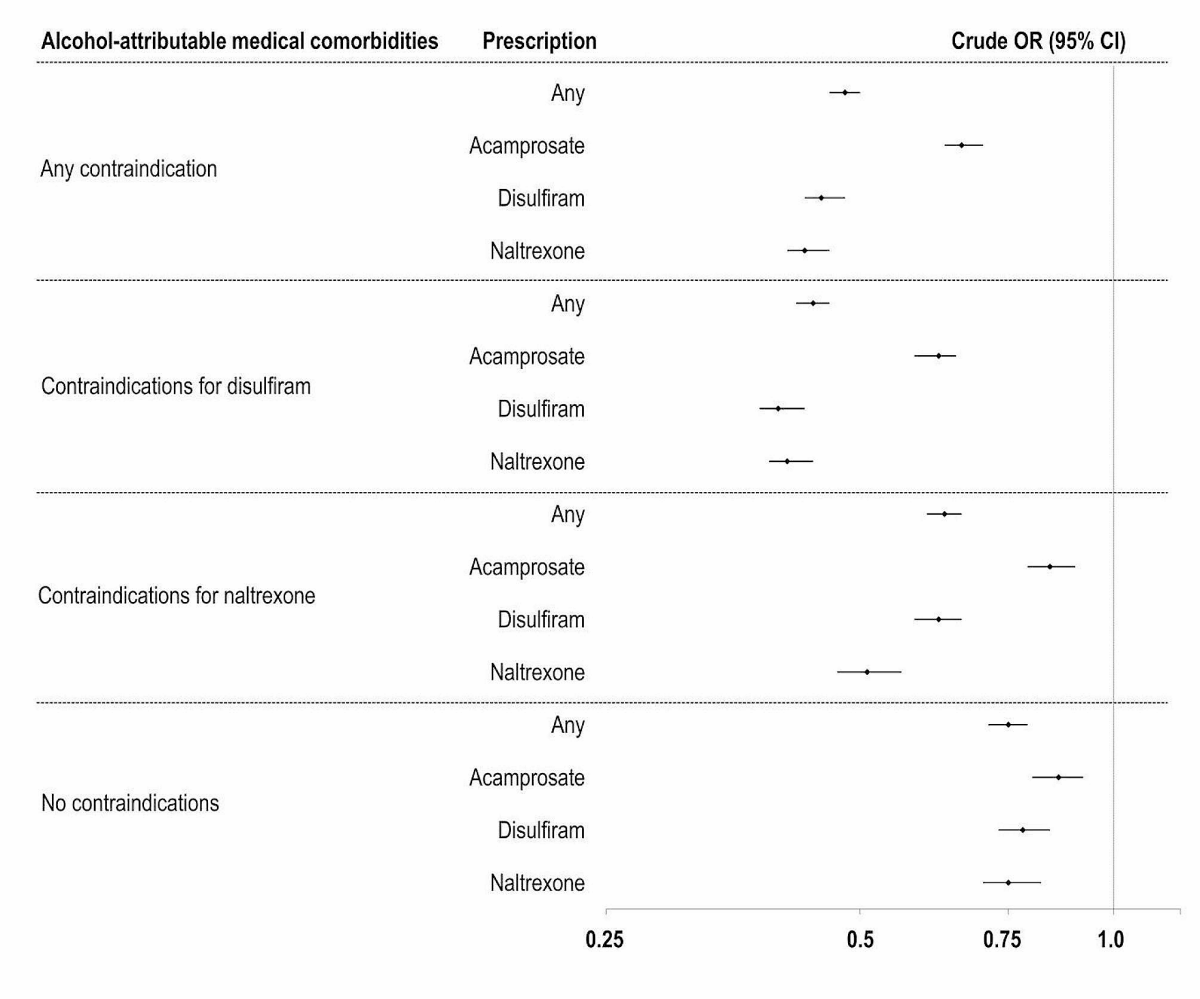
Crude odds ratios for receiving pharmacotherapy prescriptions for alcohol use disorder (AUD) by groups of comorbid diagnoses with contraindications for disulfiram and oral naltrexone treatment (*N* = 270,933). ***Note***: OR = odds ratio. CI = confidence interval. For detailed information, see Supplement Table 2

In univariate regression models, patients with both AUD diagnosis and any alcohol-attributable medical comorbidity demonstrated lower odds of filling a prescription of any type of AUD pharmacotherapy (Fig. 1 and Supplement Table 2). Comorbid cardiovascular diseases were associated with the lowest rates of receiving any prescriptions (OR = 0.41 [95% CI: 0.39–0.43]), followed by neurological (OR = 0.52 [95% CI: 0.48–0.56]) and gastrointestinal diseases (OR = 0.57 [95% CI: 0.54–0.60]). Similarly, cardiovascular, gastrointestinal, neurological diseases and cancer diagnoses were associated with the lowest rates of receiving disulfiram and naltrexone prescriptions. Moreover, diagnoses contraindicated for disulfiram and naltrexone were associated with lower odds of receiving prescriptions for all three types of pharmacotherapies (acamprosate: OR = 0.66 [95% CI: 0.63–0.70], disulfiram: OR = 0.45 [95% CI: 0.43–0.48], naltrexone: OR = 0.43 [95% CI: 0.41–0.46]) (Fig. 2 and Supplement Table 2).

In adjusted regression models, patients with an AUD diagnosis and any comorbid alcohol-attributable medical disorder filled significantly fewer prescriptions of any type of AUD pharmacotherapy compared to patients with AUD diagnosis without concurrent medical diagnosis, with the exception of infectious diseases for acamprosate, and cancer diagnoses for acamprosate (Supplement Fig. 1 and Supplement Table 3). Consistent with the univariate regression models, diagnoses with contraindication for prescription of disulfiram and naltrexone were associated with lower odds of receiving prescriptions for all three types of pharmacotherapy (acamprosate: adjusted OR = 0.81 [95% CI: 0.77–0.85], disulfiram: adjusted OR = 0.55 [95% CI: 0.52–0.59] and naltrexone: adjusted OR = 0.57 [95% CI: 0.53–0.60]) (Supplement Fig. 2 and Supplement Table 3).

The results from the sensitivity analyses using only incident AUD diagnoses did not differ from the main results and are not presented.

## Discussion

### Main findings

The aim of this study was to investigate the utilization of pharmacological treatment for AUD in specialist care among patients with comorbid alcohol-attributable medical diagnoses using a cohort of the total population in Sweden.

Our findings reveal that nearly one in four patients was diagnosed with at least one alcohol-attributable medical disorder within one year of their AUD diagnosis. The most prevalent comorbid medical diagnoses were cardiovascular and gastrointestinal diseases, followed by neurological diseases and diabetes mellitus.

Across all categories of concurrent alcohol-attributable medical comorbidities, the odds for filling any AUD pharmacotherapy prescription were consistently lower compared to AUD diagnoses without medical comorbidities. The prescription receipt rate was particularly low among AUD diagnoses with comorbid cardiovascular diseases, followed by comorbid cancer, neurological and gastrointestinal diseases — a novel finding not previously reported in the literature.

There were only small differences in the odds of filling various types of pharmacotherapies. Comorbid diagnoses which were contraindications to AUD medication were associated with lower odds for filling a prescription but did not fully explain the observed prescription gap. In particular, diagnoses with contraindications for one type of medication (e.g., disulfiram) were also associated with lower odds for receiving prescriptions for acamprosate and naltrexone. This underscores the complexity of prescribing patterns and suggests that the barriers to pharmacotherapy extend beyond contraindications.

### Prescription receipt rates for different medical disease categories

#### Gastrointestinal diseases

In 10% of all AUD diagnoses, a concurrent gastrointestinal disease was identified. Among this group, approximately 16% filled a prescription for AUD pharmacotherapy. This represents a notably higher prescription receipt rate compared to studies from the United States, where only 0.5–2.4% of patients with comorbid AUD and liver diseases received pharmacological AUD treatment [11, 17]. This disparity could be explained by variations in study design, or differences in health care systems.

Alcohol-related liver diseases, including conditions such as liver fibrosis, cirrhosis, alcoholic hepatitis and pancreatitis, constitute some of the most prominent adverse health consequences of alcohol consumption, with about half of all liver-related diseases attributed to alcohol [18]. Furthermore, effective management of alcohol consumption, particularly achieving and maintaining abstinence, plays a crucial role in increasing survival rates in liver diseases [19], whereas the persistence of alcohol consumption contributes to complications and progression [20]. AUD pharmacotherapy is considered cost-effective for patients with alcohol-related liver cirrhosis. A recent study demonstrated that medication-assisted AUD therapy in patients with alcohol-related liver cirrhosis provided greater benefits at lower costs compared to no intervention [21]. Existing evidence supports the effectiveness of AUD treatment in individuals with liver diseases and suggests treatment with acamprosate for this group [20, 22]. Similarly, interventions to reduce alcohol consumption have been shown to reduce episodes of acute pancreatitis [18, 23]. The implementation of a multidisciplinary management approach for chronic pancreatitis, involving pancreatologists and addiction specialists, has also demonstrated positive effects on patient’s drinking behaviour [24]. 

#### Cardiovascular diseases

Cardiovascular diseases constituted the second largest category of medical diseases in the present study. In 9% of all AUD diagnoses, a concurrent cardiovascular diagnosis was present. Despite their high prevalence, cardiovascular diseases showed the lowest prescription receipt rates among the medical disease categories. Specifically, odds for an individual with both cardiovascular disease and AUD filling a prescription were 59% lower in comparison to those with only AUD.

Cardiovascular diseases remain one of the leading causes of death in Europe [25]. Reduced alcohol use has been linked to improved blood pressure, with the greatest benefits observed among heavier drinkers [26]. In line with this, improvements in blood pressure have been found following treatment for alcohol dependence [27]. In a general population sample, a reduction of at least 150 g of alcohol per week among adults with heavy alcohol use was associated with lower odds for a range of self-reported cardiovascular diseases such as arteriosclerosis, angina, tachycardia, or myocardial infarction [28]. These findings emphasize the importance from a public health perspective for addressing the low prescription rate for AUD pharmacotherapy among individuals with cardiovascular diseases.

#### Other diseases

Among AUD diagnoses and either comorbid neurological disorder, diabetes mellitus, infectious diseases, or cancer, the rates of prescription receipt were similar, ranging from 15 to 18%. However, the prevalence of these comorbid diagnoses varied; approximately 4–5% of all AUD diagnoses had a comorbid neurological diagnosis or diabetes mellitus, while only 0.5-1% had a concurrent infectious disease or cancer diagnosis.

Alcohol consumption can impact health outcomes of pre-existing epilepsy by interfering with anti-epileptic drugs [29]. Moreover, alcohol consumption has been associated with reduced diabetes self-care behaviours as well as lower engagement with diabetes-related care [30, 31]. Malignancy stands out as one of the leading causes of premature death among AUD patients [32]. Additionally, in HIV/AIDS patients, alcohol use has been associated with an increased risk of transmission through sexual risk behaviour, non-compliance with antiretroviral treatment and disease progression, ultimately contributing to increased HIV/AIDS mortality [33–36]. However, similar to our findings, a recent study among American veterans identified low initiation and retention in AUD treatment among people living with HIV [37]. 

### Implications

The observed treatment gap in the present study may be attributed to factors at multiple levels, including health care systems, clinical practices, and the individual patient [8]. 

At the health care level, improved integration of specialist addiction consultation teams into the secondary level of medical care has been shown to increase initiation of pharmacotherapy [38]. For example, the implementation of an inpatient addiction medicine consultation service in a general hospital has been shown to effectively reach patients with medical diseases and AUD [39]. Interventions targeting cardiovascular and gastrointestinal settings could be particularly impactful, given they were the two largest groups of medical disorders co-occurring with AUD in this study. Additionally, mapping the current prescription practices of AUD pharmacotherapy in different health care settings, is crucial for developing healthcare services.

Moreover, development of new pharmacotherapies or repurposing of existing medications could offer additional treatment alternatives. For instance, baclofen can be prescribed to individuals with AUD and concurrent liver disease. A recent meta-analysis showed promising results for baclofen in reducing heavy drinking and increasing abstinence compared to placebo [40]. Another potential agent is varenicline [41]. However, in our study the lower odds for pharmacotherapies were not fully explained by the presence of contraindications, suggesting additional barriers beyond limited pharmacotherapy options.

On the clinician level, known barriers to prescribing AUD pharmacotherapy include perceived lack of effectiveness, time constrains and inadequate training [42–45]. Furthermore, stigmatizing attitudes among healthcare professionals towards individuals with substance use disorders are increasingly recognized as barrier to AUD treatment engagement [46, 47]. 

Barriers on the patient level are low knowledge of AUD pharmacotherapies [48], and the perception that AUD treatment is not effective [49]. Studies specifically focusing on individuals with AUD and medical comorbidities, highlight barriers such as the desire to handle alcohol-related problems independently, reluctance to abstinence-only-treatments, a perceived lack of integration between addiction care and medical care, and fear of stigmatisation [50, 51]. 

### Strength and limitations

This study used register-based data of the total population of Sweden. The registers show high level of completeness, with a low risk of selection bias. The registers have high internal validity, for example, the National Patient Register shows a positive predictive value of 85–95%, suggesting a high overlap between registered diagnoses and medical records [52]. Another strength is the use of a recognized measure of prescription rates the year following AUD diagnosis [53], contributing to a high clinical relevance of the results.

One important limitation is the absence of data on kidney diseases, which are contraindicators for acamprosate and naltrexone treatments. Furthermore, data on prescriptions other than AUD pharmacotherapy, potentially encompassing contraindications (e.g., opioid treatment), were not included. Additional methodological limitations include the measurement of medical diagnoses only following, not preceding, the AUD diagnosis, and that it was not recorded whether the medical diagnosis occurred before or after the receipt of the AUD prescription.

Also, the study solely considered filled prescriptions of AUD pharmacotherapy, capturing both the prescriber and patient behaviours. Previous Swedish research indicated an overlap of 83% between issued and filled prescriptions [54], suggesting that the presented results are largely attributed to behaviours among prescribers rather than patients. The absence of data on AUD pharmacotherapies dispensed directly at the clinic, especially for disulfiram, may lead to an underestimation of the prescriptions; however, this limitation most probably does not change the general conclusion.

Finally, register data relying on diagnoses from specialist and inpatient care represents a conservative measure of AUD in the general population, primarily capturing the more severe continuum of AUD. The dataset did not include primary care data, where up to half of all AUD and a large part of medical diagnoses are made [55]. 

The data for this study was collected between 2007 and 2015, which may be considered a limitation. However, during this period, there were no significant changes in Swedish policy or health care organization that would substantially impact the study results.

## Conclusion

There is a general utilization gap of pharmacological AUD treatment in patients with AUD and co-occurring alcohol-attributable medical disorders in specialist care, with the lowest prescription receipt rates observed in cases of cardiovascular diseases. The low prescription receipt rates were only partially attributed to concurrent medical diagnoses which were contraindicative to the pharmacotherapies. Given the associated of AUD with a wide range of medical diseases and the exacerbation of existing medical conditions, there is an urgent need to reach a larger proportion of this group with evidence-based treatments.

### Electronic supplementary material

Below is the link to the electronic supplementary material.

Supplementary Material 1

## Data Availability

The data that support the findings of this study are available from Karolinska Institute, but restrictions apply to the availability of the data, which were used under the ethical approval for the current study.

## Abbreviations

AUD: Alcohol use disorder
CI: Confidence interval
EMA: European Medicines Agency
LISA: Longitudinal Integration Database for Health Insurance and Labour Market Studies
OR: Odds ratio
RTB: Register of the Total Population
