# Supplementary material for: Pharmacotherapy for alcohol use disorder among adults with medical disorders in Sweden
Source: Addict Sci Clin Pract. 2024 May 19;19:41. doi: 10.1186/s13722-024-00471-9 (PMC11103816; doi:10.1186/s13722-024-00471-9)
Supplement: Supplementary file 1 — Supplementary Material 1 [file 13722_2024_471_MOESM1_ESM.docx]

**Supporting Information**

**Diagnostic codes according to WHO ICD-10**

Alcohol use disorder:

F10.1-F10.9

Alcohol-attributable medical diseases categories:

**Infectious Diseases:**

Tuberculosis A15–A19.9, K93.0, P37.0

HIV/AIDS B20-B24.9

Sexually transmitted diseases excluding HIV A50–A58, A60–A60.9, A63–A63.8, B63, N70–N71.9, N73–N74.8

Lower respiratory infections A48.1, A70, J11–J15.8, J16–J16.9, J20–J21.9, P23.0–P23.4

**Cancers**:

Lip and oral cavity cancer C0–C08.9, D00.00–D00.07, D10.0–D10.5, D11–D11.9, D37.01–D37.04, D37.09

Nasopharynx cancer C11–C11.9, D00.08, D10.6, D37.05

Other pharynx cancer C09–C10.9, C12–C13.9, D10.7

Oesophageal cancer C15–C15.9, D00.1, D13.0

Stomach cancer C16–C16.9, D00.2, D13.1, D37.1

Colon and rectum cancer C18–C21.9, D01.0-D01.3, D12-D12.9, D37.3–D37.5

Liver cancer C22–C22.9, D13.4

Pancreatic cancer C25–C25.9, D13.6–D13.7

Larynx cancer C32–C32.9, D02.0, D14.1, D38.0

Trachea, bronchus and lung cancer C33–C34.92, D02.1–D02.3, D14.2–D14.32, D38.1

Female breast cancer C50–C50.929, D05–D05.92, D24–D24.9, D48.6–D48.62, D49.3, N60–N60.99

Other neoplasms C17–C17.9, C3–C31.9, C37–C38.8, C4–C41.9, C47–C5, C51–C52.9, C57–C57.8, C58–C58.0, C60–C60.9, C63–C63.8, C66–C66.9, C68.0–C68.8, C69–C7, C74–C75.8, D07.4, D09.2–D09.22, D13.2–D13.39, D14.0, D15–D16.9, D28.0–D28.1, D28.7, D29.0, D30.2–D30.22, D30.4–D30.8, D31–D33.9, D35–D36, D36.1–D36.7, D37.2, D38.2–D38.5, D39.2, D39.8, D41.2–D41.3, D42–D43.9, D44.1–D44.8, D45–D45.9, D47–D47.0, D47.2–D47.9, D48.0–D48.4, D49.6, D49.81, K31.7, K62.0–K62.1, K63.5, N84.0–N84.1

**Diabetes mellitus:**

E10–E10.11, E10.3–E11.1, E11.3–E12.1, E12.3–E13.11, E13.3–E14.1, E14.3–E14.9, P70.0–P70.2

**Neurological disorders:**

Alzheimer’s disease and other dementias F00–F03.91

Epilepsy G40–G41.9

**Cardiovascular diseases**:

Hypertensive heart disease I11–I11.9

Ischaemic heart disease I20–I25.9

Cardiomyopathy B33.2–B33.24, D86.85, I40–I43.9

Atrial fibrillation and flutter I48–I48.92

Heart failure I50, I11.0, I13.0, I13.2

Ischaemic stroke G45, I63–I63.9

Haemorrhagic and other non-ischaemic stroke I60–I61.9, I62.0–I62.03

**Gastrointestinal diseases**:

Cirrhosis of the liver B18–B18.9, I98.2, K70–K70.9, K71.3–K71.51, K71.7, K72.1–K74.69, K74.9, K75.8–K76.0, K76.6–K76.7, K76.9

Gall bladder and bile duct disease K80–K83.9

Pancreatitis K85–K86.9

Other digestive diseases K57–K58, K75.2–K75.4, K90–K90.9, K92.8–K92.89

Contraindications of pharmacotherapy for alcohol use disorder:

**Disulfiram**

Alzheimer’s disease and other dementias F00–F03.91,

Hypertensive heart disease I11–I11.9

Ischaemic heart disease I20–I25.9

Cardiomyopathy B33.2–B33.24, D86.85, I40–I43.9,

Atrial fibrillation and flutter I48–I48.92

Heart failure I50, I11.0, I13.0, I13.2

Ischaemic stroke G45, I63–I63.9,

Haemorrhagic and other non-ischaemic stroke I60–I61.9, I62.0–I62.03,

Cirrhosis of the liver B18–B18.9, I98.2, K70–K70.9, K71.3–K71.51, K71.7, K72.1–K74.69, K74.9, K75.8–K76.0, K76.6–K76.7, K76.9

Liver cancer C22–C22.9, D13.4

**Naltrexone**

Cirrhosis of the liver B18–B18.9, I98.2, K70–K70.9, K71.3–K71.51, K71.7, K72.1–K74.69, K74.9, K75.8–K76.0, K76.6–K76.7, K76.9

Liver cancer C22–C22.9, D13.4

Opioid related disorders F11.1-F11.9

*Supplement Table 1 –* Total number of received acamprosate, disulfiram and naltrexone prescriptions in patients with AUD by groups of comorbid diagnoses (N = 270,933).

|  | **Acamprosate** | |  | **Disulfiram** | |  | **Naltrexone** | |  |
| --- | --- | --- | --- | --- | --- | --- | --- | --- | --- |
| **Alcohol-attributable comorbidities** | **Yes (%)** | **No (%)** | **Total (%)** | **Yes (%)** | **No (%)** | **Total** | **Yes (%)** | **No (%)** | **Total (%) **** |
| Any medical comorbidity * | 4,404 (18.0) | 59,690 (24.2) | 64,094 (23.7) | 4,550 (14.7) | 59,544 (24.8) | 64,094 (23.7) | 3,530 (14.3) | 60,564 (26.4) | 64,094 (23.7) |
| Gastrointestinal diseases | 1,987 (8.1) | 25,608 (10.4) | 27,595 (10.2) | 1,968 (6.3) | 25,627 (10.7) | 27,595 (10.2) | 1,425 (5.8) | 26,170 (10.6) | 27,595 (10.2) |
| Cardiovascular diseases | 1,431 (5.8) | 23,399 (9.5) | 24,830 (9.2) | 1,324 (4.3) | 23,506 (9.8) | 24,830 (9.2) | 1,195 (4.9) | 23,635 (9.6) | 23,830 (9.2) |
| Neurological diseases | 774 (3.2) | 11,458 (4.6) | 12,232 (4.5) | 856 (2.8) | 11,376 (4.7) | 12,232 (4.5) | 630 (2.6) | 11,602 (4.7) | 12,232 (4.5) |
| Diabetes mellitus | 686 (2.8) | 8,806 (3.6) | 9,492 (3.5) | 767 (2.5) | 8,725 (3.6) | 9,492 (3.5) | 563 (2.3) | 8,929 (3.6) | 9,942 (3.5) |
| Infectious diseases | 263 (1.1) | 3,297 (1.3) | 3,560 (1.3) | 289 (0.9) | 3,271 (1.4) | 3,560 (1.3) | 229 (0.9) | 3,331 (1.4) | 3,560 (1.3) |
| Cancers | 99 (0.4) | 1,274 (0.5) | 1,373 (0.5) | 80 (0.3) | 1,293 (0.5) | 1,373 (0.5) | 64 (0.3) | 1,309 (0.5) | 1,373 (0.5) |
| Contraindication to disulfiram and naltrexone * | 3,280 (13.4) | 45,718 (18.6) | 48,998 (18.1) | 3,272 (10.5) | 45,726 (19.1) | 48,998 (18.1) | 2,413 (9.8) | 46,585 (18.9) | 48,998 (18.1) |
| Contraindication to disulfiram | 2,828 (11.6) | 41,630 (16.9) | 44,458 (16.4) | 2,705 (8.7) | 41,753 (17.4) | 44,458 (16.4) | 2,110 (8.6) | 42,348 (17.2) | 44,458(16.4) |
| Contraindication to naltrexone | 1,897 (7.8) | 23,161 (9.4) | 25,058 (9.2) | 1,977 (6.4) | 23,081 (9.6) | 25,058 (9.2) | 1,280 (5.2) | 23,778 (9.7) | 25,058 (9.2) |
| No contraindication | 1,520 (6.2) | 17,602 (7.1) | 19,122 (7.1) | 1,783 (5.7) | 17,339 (7.2) | 19,122 (7.1) | 1,392 (5.7) | 17,730 (7.2) | 19,122 (7.1) |
| Total (%) * | 24,475 (9.0) | 246,458 (91.0) | 270,933 (100) | 31,027 (11.5) | 239,906 (88.5) | 270,933 (100) | 24,636 (9.1) | 246,297 (90.9) | 270,933 (100) |

Note: * One AUD patient can have multiple comorbid diagnoses. ** One AUD patient can fill multiple pharmacotherapy prescriptions.

Supplement Table 2 – Crude odds ratio (95% CI) for filling a pharmacotherapy prescription in patients with AUD by groups of comorbid diagnoses (N = 270,933).

| **Alcohol-attributable comorbidities** | **Any prescription** | | **Acamprosate prescription** | | **Disulfiram prescription** | | **Naltrexone prescription** | |
| --- | --- | --- | --- | --- | --- | --- | --- | --- |
|  | Crude OR | 95% CI | Crude OR | 95% CI | Crude OR | 95% CI | Crude OR | 95% CI |
| Any medical comorbidity | 0.50 | 0.48 – 0.51 | 0.67 | 0.64 – 0.70 | 0.48 | 0.46 – 0.50 | 0.48 | 0.45 – 0.50 |
| Gastrointestinal diseases | 0.57 | 0.54 – 0.60 | 0.77 | 0.72 – 0.83 | 0.54 | 0.50 – 0.58 | 0.51 | 0.47 – 0.55 |
| Cardiovascular diseases | 0.41 | 0.39 – 0.43 | 0.55 | 0.51 – 0.60 | 0.35 | 0.33 – 0.38 | 0.43 | 0.39 – 0.46 |
| Neurological diseases | 0.52 | 0.48 – 0.56 | 0.66 | 0.60 – 0.73 | 0.54 | 0.49 – 0.60 | 0.50 | 0.45 – 0.56 |
| Diabetes mellitus | 0.64 | 0.59 – 0.70 | 0.77 | 0.69 – 0.86 | 0.68 | 0.61 – 0.75 | 0.59 | 0.52 – 0.66 |
| Infectious diseases | 0.74 | 0.66 – 0.83 | 0.83 | 0.70 – 0.98 | 0.71 | 0.61 – 0.84 | 0.72 | 0.60 – 0.86 |
| Cancers | 0.49 | 0.41 – 0.60 | 0.76 | 0.58 – 0.98 | 0.42 | 0.31 – 0.56 | 0.42 | 0.30 – 0.58 |
| Contraindication to disulfiram and naltrexone | 0.48 | 0.46 – 0.50 | 0.66 | 0.63 – 0.70 | 0.45 | 0.43 – 0.48 | 0.43 | 0.41 – 0.46 |
| Contraindication to disulfiram | 0.44 | 0.42 – 0.46 | 0.62 | 0.58 – 0.65 | 0.40 | 0.38 – 0.43 | 0.41 | 0.39 – 0.44 |
| Contraindication to naltrexone | 0.63 | 0.60 – 0.66 | 0.84 | 0.79 – 0.90 | 0.62 | 0.58 – 0.66 | 0.51 | 0.47 – 0.56 |
| No contraindication | 0.75 | 0.71 – 0.79 | 0.86 | 0.80 – 0.92 | 0.78 | 0.73 – 0.84 | 0.75 | 0.70 – 0.82 |

Note: OR = odds ratio; CI = confidence interval.

Supplement Table 3 – Adjusted odds ratio (95% CI) for filling a pharmacotherapy prescription in patients with AUD by groups of comorbid diagnoses (N = 268,815).

| **Alcohol-attributable comorbidities** | **Any prescription** | | **Acamprosate prescription** | | **Disulfiram prescription** | | **Naltrexone prescription** | |
| --- | --- | --- | --- | --- | --- | --- | --- | --- |
|  | Adjusted OR | 95% CI | Adjusted OR | 95% CI | Adjusted OR | 95% CI | Adjusted OR | 95% CI |
| Any medical comorbidity | 0.61 | 0.59 – 0.63 | 0.79 | 0.75 – 0.83 | 0.57 | 0.55 – 0.60 | 0.61 | 0.58 – 0.64 |
| Gastrointestinal diseases | 0.64 | 0.61 – 0.67 | 0.86 | 0.80 – 0.92 | 0.58 | 0.54 – 0.62 | 0.60 | 0.56 – 0.65 |
| Cardiovascular diseases | 0.55 | 0.52 – 0.59 | 0.71 | 0.65 – 0.76 | 0.48 | 0.44 – 0.52 | 0.60 | 0.55 – 0.65 |
| Neurological diseases | 0.64 | 0.59 – 0.69 | 0.78 | 0.70 – 0.86 | 0.66 | 0.60 – 0.73 | 0.63 | 0.56 – 0.71 |
| Diabetes mellitus | 0.75 | 0.69 – 0.81 | 0.87 | 0.78 – 0.97 | 0.77 | 0.69 – 0.85 | 0.72 | 0.64 – 0.81 |
| Infectious diseases | 0.80 | 0.71 – 0.90 | 0.87 | 0.74 – 1.04 | 0.82 | 0.69 – 0.98 | 0.72 | 0.60 – 0.86 |
| Cancers | 0.60 | 0.49 – 0.73 | 0.85 | 0.65 – 1.10 | 0.56 | 0.41 – 0.75 | 0.49 | 0.35 – 0.67 |
| Contraindication to disulfiram and naltrexone | 0.60 | 0.58 – 0.63 | 0.81 | 0.77 – 0.85 | 0.55 | 0.52 – 0.59 | 0.57 | 0.53 – 0.60 |
| Contraindication to disulfiram | 0.57 | 0.55 – 0.59 | 0.76 | 0.72 – 0.81 | 0.50 | 0.47 – 0.53 | 0.55 | 0.52 – 0.59 |
| Contraindication to naltrexone | 0.70 | 0.67 – 0.74 | 0.94 | 0.88 – 1.01 | 0.65 | 0.60 – 0.70 | 0.60 | 0.56 – 0.66 |
| No contraindication | 0.79 | 0.75 – 0.83 | 0.89 | 0.82 – 0.95 | 0.82 | 0.76 – 0.88 | 0.81 | 0.75 – 0.87 |

Note: Adjusted for sex, age, income, education, family constellation, domicile and country of birth. OR = odds ratio. CI = confidence interval.

**Supplementary Figure 1** – Adjusted odds ratios for filled prescription of AUD pharmacotherapy by groups of comorbid medical diagnoses (*N* = 268,815).


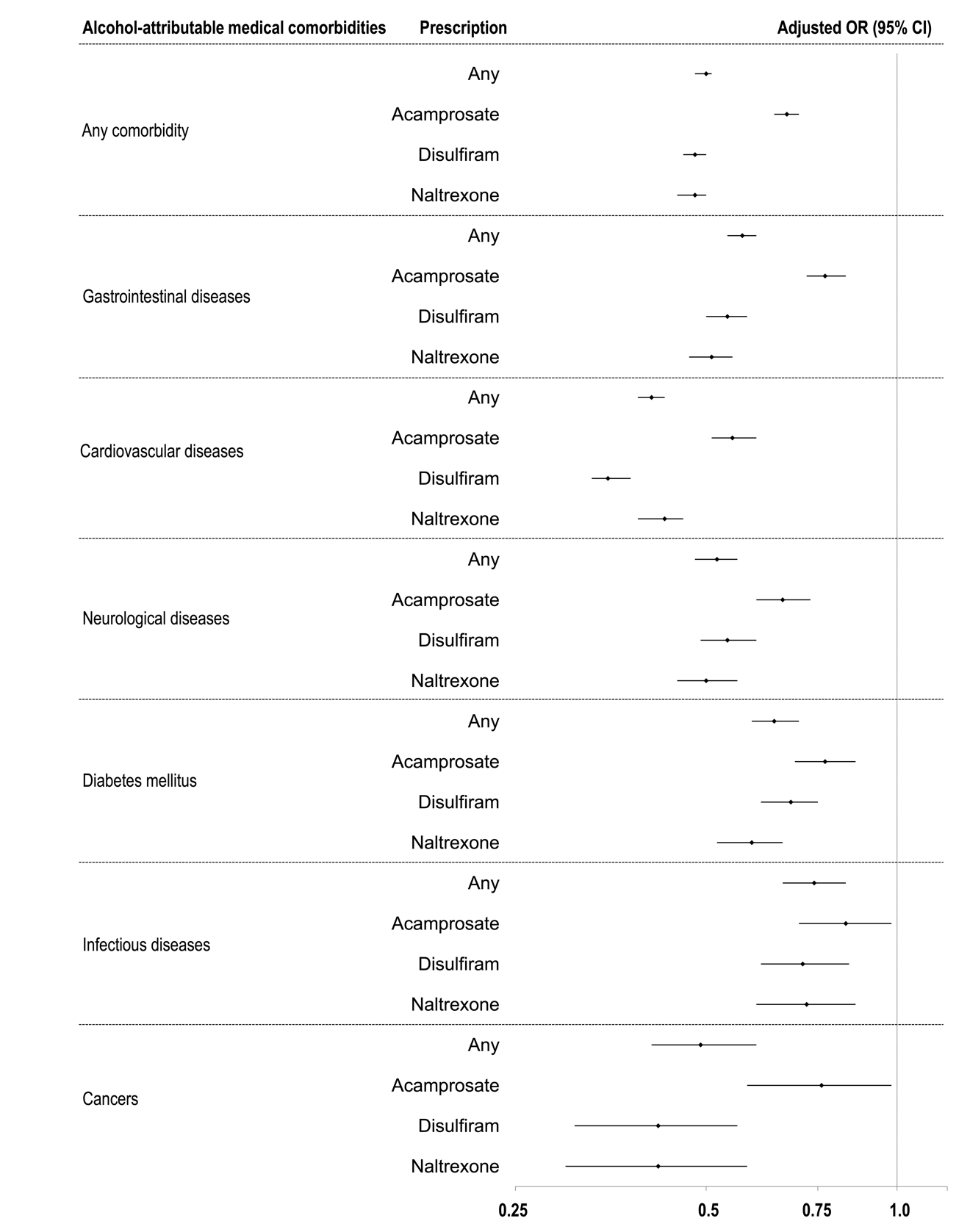


Note: Adjusted for sex, age, income, education, family constellation, domicile and country of birth. OR = odds ratio. CI = confidence interval. For detailed information, see Supplement Table 3.

**Supplementary Figure 2** – Adjusted odds ratios filled prescription of AUD pharmacotherapy by groups of comorbid diagnoses with contraindications to disulfiram and naltrexone (N = 268,815).


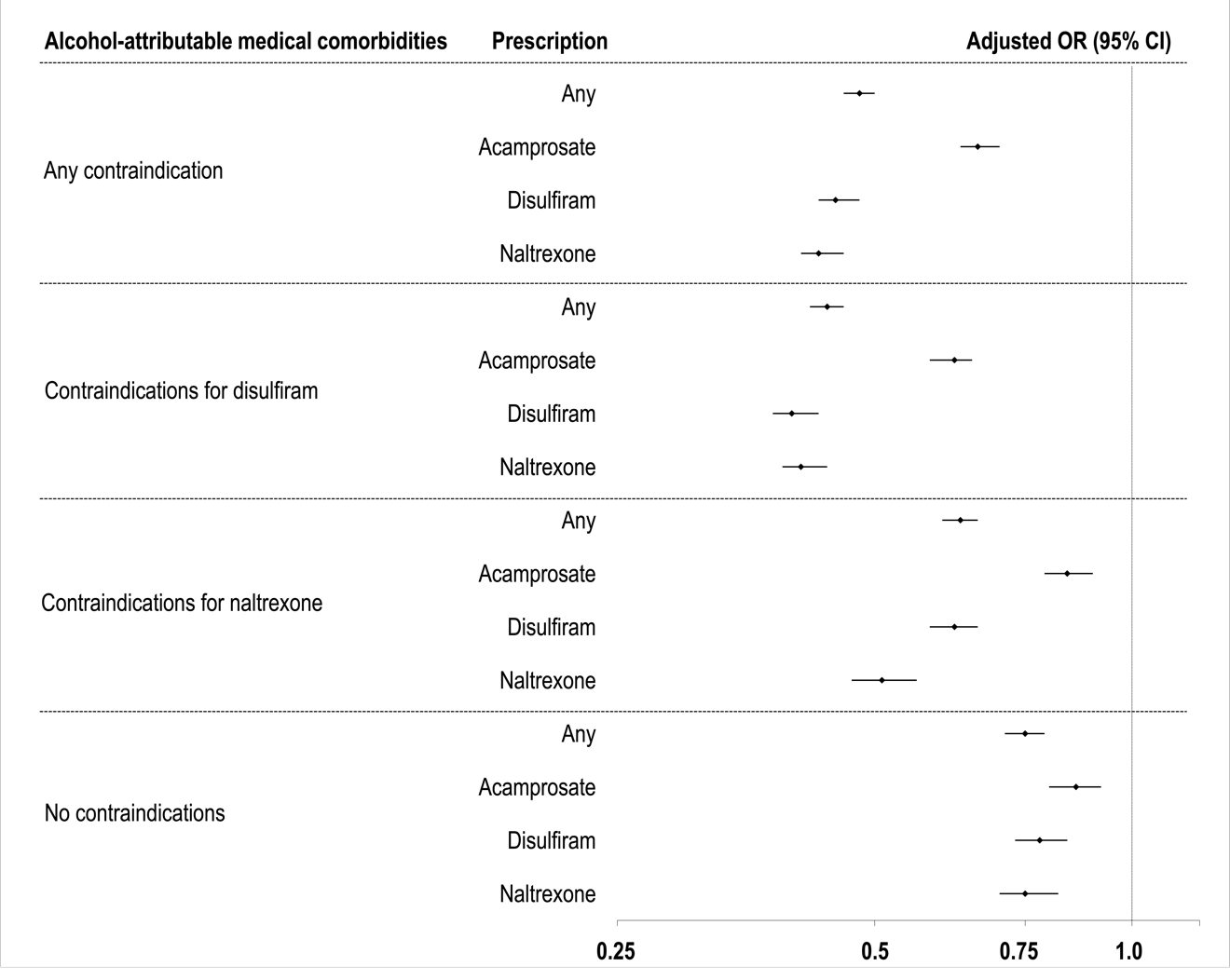


Note: Adjusted for sex, age, income, education, family constellation, domicile and country of birth. OR = odds ratio. CI = confidence interval. For detailed information, see Supplement Table 3.
